# Optimal Passive Source Localization for Acoustic Emissions

**DOI:** 10.3390/e23121585

**Published:** 2021-11-27

**Authors:** Carlos A. Prete, Vítor H. Nascimento, Cássio G. Lopes

**Affiliations:** Department of Electronic Systems Engineering, University of São Paulo, São Paulo 3566-590, Brazil; vitnasci@usp.br (V.H.N.); cassio.lopes@usp.br (C.G.L.)

**Keywords:** acoustic emissions, time-of-arrival, source localization

## Abstract

Acoustic emission is a non-destructive testing method where sensors monitor an area of a structure to detect and localize passive sources of elastic waves such as expanding cracks. Passive source localization methods based on times of arrival (TOAs) use TOAs estimated from the noisy signals received by the sensors to estimate the source position. In this work, we derive the probability distribution of TOAs assuming they were obtained by the fixed threshold technique—a popular low-complexity TOA estimation technique—and show that, if the sampling rate is high enough, TOAs can be approximated by a random variable distributed according to a mixture of Gaussian distributions, which reduces to a Gaussian in the low noise regime. The optimal source position estimator is derived assuming the parameters of the mixture are known, in which case its MSE matches the Cramér–Rao lower bound, and an algorithm to estimate the mixture parameters from noisy signals is presented. We also show that the fixed threshold technique produces biased time differences of arrival (TDOAs) and propose a modification of this method to remove the bias. The proposed source position estimator is validated in simulation using biased and unbiased TDOAs, performing better than other TOA-based passive source localization methods in most scenarios.

## 1. Introduction

Acoustic emission testing is a non-destructive testing method used to detect several kinds of faults in structures such as piping, bridges and aerospace structures. Its main advantages over other non-destructive testing methods are the high sensibility, the wide coverage area and the possibility of monitoring structures in real time [1,2,3,4,5,6]. In the acoustic emission framework, sensors are spread over the surface to be monitored, and elastic waves emitted by passive sources (such as cracks or rivets under stress) propagate through the medium and are detected by these sensors. The segment of signal acquired by a sensor that is detected as a wave is called a *hit*. In order to reduce memory requirements, the sampled signals are usually not saved, only the estimated times of arrival (TOAs) are recorded and later used to estimate the source position.

Many TOA estimation algorithms are presented in the literature [7,8,9,10], but the fixed threshold method [11,12,13] is a very popular technique due to its simplicity and low complexity. Defining $r_{i}\left[n\right]$ as the signal sampled with period *T* by the *i*-th sensor at instant t=nT, this method estimates the TOA $t_{i}$ by comparing the absolute value of the signal to a fixed threshold *K*:

(1) $$t_{i}=n_{i}T,\;n_{i}=\min_{n}\left\{n:\;\right|r_{i}\left[n\right]\left|\geq K\right\}.$$

Thus, the estimated TOA is the first instant the absolute value of the signal crosses the threshold. The value of K should be high enough to be larger than the noise level most of the time, but not too high so that hits will be missed.

There are many approaches in the literature to estimate the source position given the TOAs [14,15,16,17,18,19,20], usually based on the minimization of different choices of cost function that may receive as input the measured TOAs or the time-differences of arrival (TDOAs, defined as the difference of TOAs measured by different sensors). One of the main contributions of this paper is to compare different cost functions and show that they do not usually lead to optimal estimates of source localization in general, except in the particular situation of small noise variance. We concentrate here in the isotropic case, i.e., we assume that the wave velocity is known and independent of the direction, but there are algorithms that are applicable to the anisotropic and unknown velocity case [14,21,22].

Passive source localization algorithms based on TOAs usually assume a Gaussian distribution for the uncertainties in the TOA estimates and a zero-mean Gaussian distribution for TDOA estimates [20,21,22,23,24,25,26]. We prove theoretically that this assumption holds approximately for the threshold method in the case of low noise. In a more general setting, the TOA distribution depends on the method used to estimate it, and we prove in Section 3 that for larger noise variances the fixed threshold method produces TOAs that can be modeled as a mixture of Gaussians (see also [27]). Furthermore, TDOAs estimated by the fixed threshold method may present a large bias since signals reach sensors with different amplitudes due to attenuation (see Section 4 and [27]).

**Figure 1:**
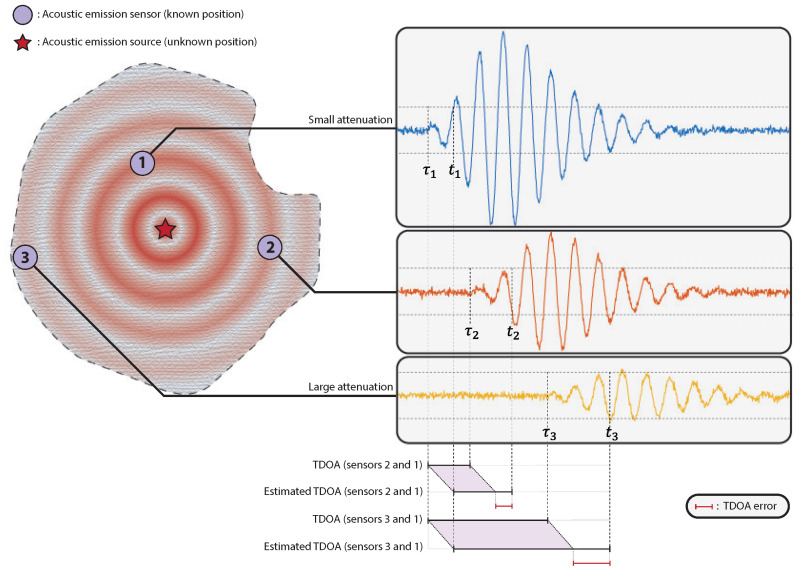
Illustration of the fixed threshold method. TOAs are obtained by comparing waveforms received at sensors with a fixed threshold. The symbols $\tau_{i}$ and $t_{i}$ represent, respectively, the true and estimated TOA for sensor *i*. Because the signals sampled by different sensors usually have different amplitudes due to attenuation, the fixed threshold method produces biased TDOAs.

The fixed threshold method only works if the threshold *K* is sufficiently higher than the noise level so that noise has a low probability of triggering the threshold without an incoming wave. However, choosing a high value for *K* increases the TOA bias, given by the difference between the expected value of the estimated and the actual time of arrival of the wave. If all sensors received hits with similar amplitudes, the TOA bias would be constant accross sensors, and there would be no TDOA bias. However, because sensors lie at different distances from the acoustic emission source, they usually receive hits with different amplitudes due to attenuation. As such, TOA bias varies accross sensors, generating a bias in TDOA estimates, which may bias the position estimate. An illustration of the fixed threshold method and the bias in TOA and TDOA estimates is presented in Figure 1.

The contributions of this paper are:

- To derive the probability distribution for TOA estimates obtained through the fixed threshold method. We show that the TOAs can be well approximated by a mixture of Gaussian distributions when (as is usually the case) the sensor has a bandpass frequency response, such that the observed signals may be well described by a model of the form
  (2) $$s\left(t\right)=e\left(t\right)\sin\left(2\pi f_{0}t+\phi \right),$$
  where e(t) is a low-pass envelop function and $f_{0}$ is the peak of the sensor frequency response. The mixture of Gaussians is a reasonable approximation if the sampling rate $f_{s}$ satisfies $f_{s}\gg 4f_{0}$. We also show that the Gaussian distribution usually assumed is a good approximation in the low-noise regime.
- To propose a TOA estimation method that generates unbiased Time Differences of Arrival (TDOAs) for structures where all frequencies propagate in the medium with the same velocity.
- To derive the Cramér–Rao lower bound for the source position assuming TOAs follow a mixture of Gaussian distributions.
- To derive the optimal TOA-based source position estimator on a surface (that is, the estimator whose covariance matrix is the inverse of the Fisher information matrix) assuming TOAs follow a mixture of Gaussian distributions whose parameters are known given the source position. A procedure to estimate these parameters from noisy signals is also presented. The optimal estimator is tested in simulation in several scenarios and compared with other passive source localization methods based on TOAs.
- To show that the optimal estimator reduces to one of the algorithms available in the literature if the TOA estimates are unbiased and the measurement noise is sufficiently small, in which case TOAs are approximately Gaussian-distributed.

**Notation:** We denote the expected value operator as E{·}, the probability of an event A as P{A}, the multivariate normal distribution with mean vector μ and covariance matrix C as N(μ,C), the covariance matrix of a random vector x as cov(x), the trace of matrix A as tr{A}. $\mathrm{diag}(a_{1},\cdots ,a_{N})$ is the diagonal matrix A such that $A_{ii}=a_{i}$, $1_{A}\left(x\right)$ is the indicator function, equal to 1 if x∈A and 0 otherwise, and $A^{c}$ is the complement of the set A. Vectors and matrices are written in boldface, and the derivative of the *k*-th element of a vector v with respect to *x* is denoted as $v_{k,x}$.

## 2. Source Localization Methods Based on TOAs

Hits identified at different sensors with similar TOAs are grouped into a single *event* related to a possible source [12]. Assuming the grouping is correct, the source position can be estimated by solving the system of equations:

(3) $$t_{k}=t+\tau_{k}\left(x\right),\;k=1,2,\cdots ,N,$$

where $t_{k}$ is the TOA at sensor *k*, *N* is the number of sensors, *t* is the instant the event occurred (an unknown variable) and $\tau_{k}\left(x\right)$ is the time the wave on the surface under test takes to propagate from the tentative source position $x={\left[\begin{array}{cc} x & y \end{array}\right]}^{T}$ to the position $x_{k}$ of the *k*-th sensor assuming the propagation is isotropic with known velocity *c*:

(4) $$\tau_{k}\left(x\right)=\frac{1}{c}∥x-x_{k}∥.$$

The system of Equations (3) has three unknowns (x,y and *t*) and *N* equations, thus it is overdetermined for N>3 unless there are co-linear sensors. Since due to noise the system usually has no solution, the problem is recast as an optimization problem using an appropriate cost function to obtain an approximate solution. Three popular cost functions for TOA-based source localization are (5), (8) and (9) below [12,20,28]. The quality of the position estimates depends on the choice of the cost function, as well as on the distribution of the uncertainty in the TOA estimates $t_{i}$.

The least squares solution of (3) is obtained by minimizing the cost function $J_{\mathrm{TOA}}\left(x,t\right)$, defined as

(5) $$J_{\mathrm{TOA}}\left(x,t\right)=\sum_{k=1}^{N}{\left(t_{k}-t-\tau_{k}\left(x\right)\right)}^{2}.$$

$J_{\mathrm{TOA}}\left(x,t\right)$ is a generalization of the cost function $J_{\mathrm{TOA}}^{\prime }\left(x\right)=c^{2}\sum_{k=1}^{N}{\left(t_{k}-\tau_{k}\left(x\right)\right)}^{2}$ [23] that is used in applications where the instant of emission of the wave $t^{*}$ is known, in which case it is common to use $t^{*}=0$ without loss of generality. In our application, $t^{*}$ is unknown and it must be taken into account to localize the source. Fortunately, it is possible to obtain a cost function equivalent to (5) that only depends on x by solving for *t* as a function of x. In fact, setting $\frac{\partial }{\partial t}J_{\mathrm{TOA}}\left(x,t\right)=-2\sum_{k=1}^{N}\left(t_{k}-t-\tau_{k}\left(x\right)\right)$ to zero and isolating *t* yields $t=\frac{1}{N}\sum_{k=1}^{N}\left(t_{k}-\tau_{k}\left(x\right)\right)$.

Hence, minimizing $J_{\mathrm{TOA}}\left(x,t\right)$ is equivalent to minimizing:

(6) $$J_{\mathrm{TOA}}\left(x\right)=\sum_{k=1}^{N}{\left(t_{k}-\tau_{k}\left(x\right)-t\left(x\right)\right)}^{2},$$

where $t\left(x\right)=\frac{1}{N}\sum_{k=1}^{N}\left(t_{k}-\tau_{k}\left(x\right)\right)$. Another approach to approximately solve (3) is to subtract the first equation from the others to eliminate *t*, yielding a modified system of N−1 equations in two unknowns whose *k*-th equation is:

(7) $$t_{k}-t_{1}=\tau_{k}\left(x\right)-\tau_{1}\left(x\right),\;2\leq k\leq N.$$

The least squares solution of (7) is obtained by minimizing the cost function $J_{\mathrm{TDOA}}\left(x\right)$ below, which only depends on the measured time differences of arrival (TDOAs) $t_{k}-t_{1}$ [12,29]:

(8) $$J_{\mathrm{TDOA}}\left(x\right)=\sum_{k=2}^{N}{\left(t_{k}-t_{1}-\left(\tau_{k}\left(x\right)-\tau_{1}\left(x\right)\right)\right)}^{2}.$$

It is also possible obtain an approximate solution to (3) using a constrained least squares approach, as done in [28]. Without loss of generality, consider that sensor 1 is positioned at (0,0). Rewriting each equation from (7) as ${\left(t_{k}-t_{1}+\tau_{1}\left(x\right)\right)}^{2}=\tau_{k}{\left(x\right)}^{2}$, substituting (4), expanding the squares and performing simple algebraic manipulations, we obtain $d_{k}∥x∥+x_{k}^{T}x=\frac{{∥x_{k}∥}^{2}-d_{k}^{2}}{2}$, where $d_{k}=c\left(t_{k}-t_{1}\right)$ is the measured range difference between sensors *k* and 1.

In order to find an approximate solution for this over-determined system of equations, Ref [28] defines the auxiliary unknown variable $z={\left[\begin{array}{cc} ∥x∥ & x \end{array}\right]}^{T}$, which is estimated by defining the cost function $J_{CLS}\left(z\right)$ (where the acronym ’CLS’ stands for ’constrained least squares’) as

(9) $$J_{CLS}\left(z\right)={∥Az-b∥}^{2},$$

where $A=\left[\begin{array}{cc} d_{2} & x_{2}^{T}\\ \vdots & \vdots \\ d_{N} & x_{N}^{T} \end{array}\right]$ and $b=\frac{1}{2}{\left[\begin{array}{ccc} {∥x_{2}∥}_{2}^{2}-d_{2}^{2} & \cdots & {∥x_{N}∥}_{2}^{2}-d_{N}^{2} \end{array}\right]}^{T}$, and solving the constrained optimization problem:

(10) $$\hat{z}=\arg\min_{z}J_{CLS}\left(z\right)\;s.t.\;\left\{\begin{array}{c} z_{1}^{2}-z_{2}^{2}-z_{3}^{2}=0\\ z_{1}>0 \end{array}\right.,$$

in which we denote by $z_{i}$ the *i*-th element of vector z. A low-complexity method for obtaining an approximate solution for (10) is proposed in [28], but since the focus of this paper is on the quality of the estimators, in our simulations we solve (10) directly.

## 3. TOA Probability Distribution

In [27], the probability mass function (pmf) for TOAs obtained by the fixed threshold algorithm was derived considering the measurement noise to be white. In this section, we extend that result, deriving the TOA pmf for non-white noise, and obtaining a simplified expression in the case where the noise is white.

### 3.1. TOA Pmf for Correlated Noise

Let us model the signal r[n] sampled by a sensor as the sum of a deterministic component s[n] and a zero-mean noise w[n], that is, r[n]=s[n]+w[n]. To simplify the notation, the index *i* representing the sensor is omitted.

The fixed threshold method estimates the TOA as the first instant the signal |r[n]| crosses the threshold. Thus, denoting as p[n] the probability of the estimated TOA being the instant *n*, we have:

(11) p[n]=P{|r[n]|≥Kand|r[m]|<K∀m<n}.

Note that the threshold level should be adjusted so that the noise cannot trigger the threshold by itself [11]. Assume then that the threshold is well adjusted (i.e., $P\{|w_{k}\left[m\right]|\geq K\}\approx 0\;\forall m$). Then, |r[n]|≥K if and only if:

(12) $$w\left[n\right]\mathrm{sgn}\left(s[n]\right)\geq q_{n},\;q_{n}\overset{\mathrm{def}}{=}K-\left|s\left[n\right]\right|.$$

Let us prove (12): If |r[m]|≥K, then sgn(r[n])=sgn(s[n]) because w[n]∈[−K,+K]. Thus,

(13) |r[n]|=(s[n]+w[n])sgn(r[n])=|s[n]|+w[n]sgn(w[n])≥K,

leading to:

(14) $$w\left[n\right]\mathrm{sgn}\left(s\left[n\right]\right)\geq q_{n}.$$

To prove the converse of (12), assume that w[n]sgn(s[n])≥K−|s[n]|. If s[n]≥0, this implies s[n]+w[n]≥K, thus $w\left[n\right]\mathrm{sgn}\left(s\left[n\right]\right)\geq q_{n}$. On the other hand, if s[n]<0, we have −w[n]−s[n]=w[n]sgn(s[n])+|s[n]|≥K, also leading to $w\left[n\right]\mathrm{sgn}\left(s\left[n\right]\right)\geq q_{n}$.

Our goal is to write (11) in terms of the noise joint cumulative distribution. For this, define the intervals $I_{m}=\;]-\infty ,q_{m}[$ and the modified noise $\tilde{w}\left[m\right]=\mathrm{sgn}\left(s\left[m\right]\right)w\left[m\right]$ for 0≤m≤n. Using these definitions, we conclude from (12) that |r[n]|<K if and only if $\tilde{w}\left[n\right]<q_{m}$, thus p[n] is the probability of $\left(\tilde{w}\left[n\right],\tilde{w}\left[n-1\right],\cdots ,\tilde{w}\left[0\right]\right)$ belonging to $I_{n}^{c}\times I_{n-1}\times \cdots \times I_{0}$. Define the vector of noise samples $w\left[n\right]={\left[\begin{array}{cccc} w[0] & w[1] & \cdots & w[n] \end{array}\right]}^{T}$. We consider that the joint noise pdf $f_{w[n]}\left(w_{0},\cdots ,w_{n}\right)$ is an even function with respect to each variable, that is, $f_{w[n]}\left(w_{0},\cdots ,w_{j},\cdots ,w_{n}\right)$ = $f_{w[n]}\left(w_{0},\cdots ,-w_{j},\cdots ,w_{n}\right)$ for all *j* (e.g., a zero-mean Gaussian distribution). In this case, $\left(\tilde{w}\left[n\right],\cdots ,\tilde{w}\left[0\right]\right)$ has the same distribution as w[n]. Hence, we have $p\left[n\right]=P\{w\left[n\right]\in I_{n}^{c}\times I_{n-1}\times I_{n-2}\times \cdots \times I_{0}\}$, which can be expanded into:

(15) $$\begin{array}{cc} p[n] & =\int_{I_{0}\times \cdots \times I_{n-1}\times I_{n}^{c}}f_{w[n]}\left(w_{0},\cdots ,w_{n}\right)dw_{0}\cdots dw_{n}\\ \; & =\int_{-\infty }^{+\infty }\int_{I_{0}\times \cdots \times I_{n-1}}f_{w[n]}\left(w_{0},\cdots ,w_{n}\right)dw_{0}\cdots dw_{n}\\ \; & -\int_{I_{0}\times \cdots \times I_{n-1}\times I_{n}}f_{w[n]}\left(w_{0},\cdots ,w_{n}\right)dw_{0}\cdots dw_{n}. \end{array}$$

As the second integral in (15) is equal to the joint cumulative distribution of w[n] evaluated at $\left(q_{0},q_{1},\cdots ,q_{n}\right)$ and the first integral is the cumulative distribution of w[n−1] evaluated at $\left(q_{0},q_{1},\cdots ,q_{n-1}\right)$, we obtain:

(16) $$p\left[n\right]=F_{w[n-1]}\left(q_{0},\cdots ,q_{n-1}\right)-F_{w[n]}\left(q_{0},\cdots ,q_{n}\right),$$

where $F_{w[n]}\left(\cdot \right)$ is the joint cumulative distribution of w[n].

### 3.2. TOA Pmf for Independent and Identically Distributed Noise

If the noise is an independent and identically distributed (i.i.d.) stochastic process, a much simpler expression for (16) can be obtained. In this case, denoting the cumulative density function (cdf) of a single noise sample as $F_{W}\left(w\right)$, the joint cumulative distribution $F_{w[n]}\left(w\right)$ can be written as the product of the cdfs of each noise sample, that is, $F_{w[n]}\left(w\right)=\prod_{\ell =0}^{n}F_{W}\left(w_{\ell }\right)$, and thus:

(17) $$p\left[n\right]=\left(1-F_{W}\left(q_{n}\right)\right)\prod_{\ell =0}^{n-1}F_{W}\left(q_{\ell }\right),\;q_{\ell }=K-\left|s\left[\ell \right]\right|.$$

Note that $q_{n}$ and p[n] depend on the noiseless signal s[n], which is unknown in practical applications. In Section 5.3, $q_{n}$ is estimated using the noisy signal r[n] instead of s[n], and (17) is used to build a source position estimator.

### 3.3. Approximating the TOA Pdf as a Gaussian Mixture

Many authors consider that TOAs follow a Gaussian distribution to derive passive source localization methods [23,24,25,26], but (17) is not only discrete, but may also be multimodal. In this section, we investigate in which cases the Gaussian distribution is a good approximation to the TOA pmf (17). We show via an example that, for high sampling rate and small noise variance, TOAs can be well approximated by a Gaussian distribution, and for high sampling rate and high noise variance they can be approximated by a mixture of Gaussian distributions. This approximation is exploited in Section 5.1, where the expression for the optimal TOA-based estimator for TOAs distributed according to a mixture of Gaussian distributions is derived.

Using a sampling rate of 10MHz (sampling period T=0.1μs), the received signal was generated following (2) as $r\left[n\right]=\sin^{2}\left(\frac{\pi (n-1)}{1000}\right)\sin\left(\frac{2\pi (n-1)}{67}\right)+w\left[n\right]$ (a von Hann window modulated with a frequency of $f_{0}=\frac{1}{67\;\mu s}\approx 150\;\mathrm{kHz}$, a common resonance frequency for piezoelectric sensors used in acoustic emission), where w[n] is a Gaussian white noise with standard deviation $\frac{\mu sK}{5}=0.06$, and K=0.3 is the threshold. We then applied the fixed threshold method to r[n] to obtain the experimental TOAs and generate the empirical TOA pmf and compare with the theoretical expression (17).

**Figure 2:**
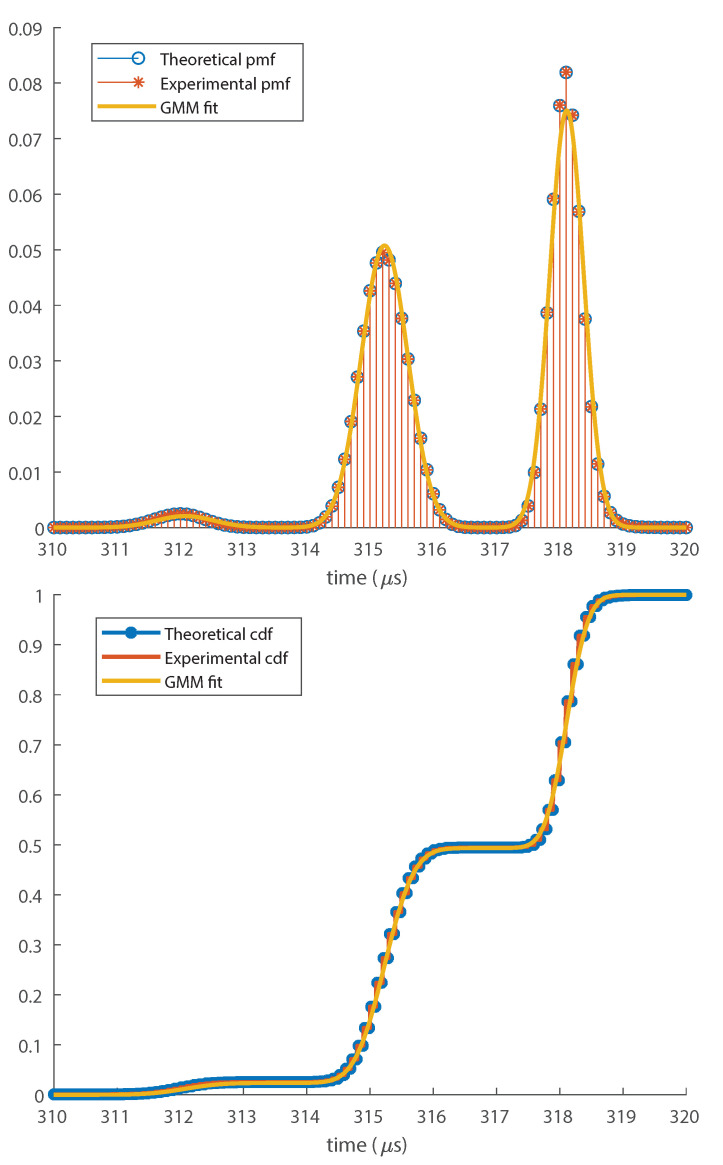
TOA pmf (top) and cdf (bottom) for high noise level compared with the fitted Gaussian mixture distribution. GMM, Gaussian mixture model.

The obtained TOA pmf and cdf for this hit is shown in Figure 2. The pmf p[n] was fitted into a Gaussian Mixture Model (GMM), leading to the pdf $p_{\mathrm{GMM}}\left(t\right)$. The details of the fitting technique we used are described in Appendix C. The cdf of the fitted distribution was plotted along with the TOA cdf, and in order to allow the comparison between the fitted pdf $p_{\mathrm{GMM}}\left(t\right)$ and p[n], we plotted $T\times p_{\mathrm{GMM}}\left(t\right)$ along with p[n].

We conclude from Figure 2 that the TOA distribution is well approximated by a GMM. If the noise level is high enough, the threshold may be triggered in different oscillation periods of the signal, creating secondary lobes spaced by the oscillation period of |r[n]| (half the oscillation period of r[n]), explaining the different Gaussian components of p[n]. On the other hand, if the noise were small, p[n] would present a single lobe, reducing to a Gaussian distribution.

Since we are approximating a discrete probability distribution by a continuous distribution, this approximation is only reasonable if the sampling rate is high enough so that each oscillation period of |r[n]| contains multiple samples, leading to multiple samples for each lobe of p[n]. Furthermore, each lobe of p[n] should correspond to a single oscillation period of |r[n]|, thus there must be at least one sample below the threshold between oscillation periods of |r[n]|. Hence, the sampling rate $f_{s}$ must satisfy $f_{s}\gg 4f_{0}$, where $f_{0}$ is the carrier frequency. Nevertheless, if the sampling rate is small enough so that each lobe of p[n] contains only one sample, p[n] may be approximated by a mixture of Gaussian distributions with very small variances. It is worth noting that if both the sampling rate and the noise are small, TOAs may become deterministic, in which case any unbiased estimator produces the same outcome.

## 4. Estimating Unbiased TDOAs

In general, sensors receive hits generated by the same source with different amplitudes, since the distance of propagation is different for each sensor and waves attenuate as they propagate. Moreover, the attenuation may be frequency-dependent, so that the signal waveforms may differ at each sensor. Because of the difference of attenuation among sensors, the lag between the TOA estimated by the fixed threshold method and the actual time of arrival is not the same for all sensors (that is, $E\left\{t_{i}-t_{j}\right\}\neq \tau_{i}-\tau_{j}$ even for noise variance tending to zero). As such, the fixed threshold method produces biased TDOA estimates, which will lead to biased position estimates. The fixed threshold method thus only produces unbiased estimates in scenarios with small attenuation difference between hits received by sensors.

We now derive a low-complexity method to obtain unbiased TDOAs in the noiseless case that consists of using different thresholds for each sensor, extending the fixed threshold method to a scenario where attenuation does not necessarily produce biased TDOA estimates. For this, we assume that waveforms at different sensors have the same shape with different amplitudes. In this model, the analog signals received by sensors $r_{i}\left(t\right)$ are attenuated and delayed versions of each other:

(18) $$r_{i}\left(t\right)=a_{i}\psi \left(t-\tau_{i}\right),$$

$a_{i}$ is a known function of the distance between the source and the *i*-th sensor (denoted as $d_{i,s}$) that models the attenuation and ψ(t) is an auxiliary function that controls the shape of the received signals. In Section 6, we use $a_{i}=\frac{e^{-\alpha d_{i,s}}}{\sqrt{d_{i,s}}}$, where the term $e^{-\alpha d_{i,s}}$ models the energy loss and the term $\frac{1}{\sqrt{d_{i,s}}}$ is due to the wave dispersion in the 2D medium [30]. Note that (18) assumes the frequency response of all sensors to be similar. Furthermore, if the structure generates reflections of the source signal, then (18) may be valid only for instants *t* smaller than the instant the first reflection arrives at the sensor.

Define $E_{i}=\sqrt{\frac{1}{M}\sum_{n}r_{i}^{2}\left[n\right]}$ as the RMS value of the hit corresponding to the *i*-th sensor, and $\beta_{i}=\frac{\sum_{k=1}^{N}E_{k}}{E_{i}}$. We propose to obtain TOAs in real time by comparing $r_{i}\left[n\right]$ with the modified threshold $K_{i}=\frac{K}{\beta_{i}}$ instead of using a fixed threshold. This way, the normalized threshold $K_{i}$ is used to estimate the TOAs given that a hit was detected.

We now show that if signals follow the propagation model (18), this procedure produces unbiased TDOAs in the noiseless case. Since $r_{i}\left[n\right]=r_{i}\left(nT\right)=a_{i}\psi \left(nT-\tau_{i}\right)$, we have $E_{i}=\sqrt{\frac{1}{M}\sum_{n}a_{i}^{2}\psi^{2}\left(nT-\tau_{i}\right)}=a_{i}E_{0}$, where $E_{0}=\sqrt{\frac{1}{M}\sum_{n}\psi^{2}\left(nT-\tau_{i}\right)}$. Hence, $\beta_{i}=\frac{\sum_{k=1}^{N}a_{k}E_{0}}{a_{i}E_{0}}=\frac{\sum_{k=1}^{N}a_{k}}{a_{i}}$.

The estimated TOA is $t_{i}=T\min_{n}\left\{n:\;\right|r_{i}\left[n\right]|\geq \frac{K}{\beta_{i}}\}=T\min_{n}\{n:\;\beta_{i}a_{i}|\psi \left(nT-\tau_{i}\right)\left|\geq K\right\}$, and substituting $\beta_{i}=\frac{\sum_{k=1}^{N}a_{k}}{a_{i}}$, we obtain:

(19) $$\begin{array}{cc} t_{i} & =T\min_{n}\{n:\;|\psi \left(nT-\tau_{i}\right)\left|\sum_{k=1}^{N}a_{k}\geq K\right\}\\ \; & =T\min_{n}\left\{n:\;|\psi \left(nT\right)\right|\sum_{k=1}^{N}a_{k}\geq K\}-\tau_{i}. \end{array}$$

The term $\left|\psi \left(nT\right)\right|\sum_{k=1}^{N}a_{k}$ in (19) does not depend on *i*, thus the TDOA between hits measured by sensors *i* and *j* is $t_{i}-t_{j}=\tau_{i}-\tau_{j}$, which is an unbiased TDOA measurement in the noiseless case.

Note that even though the waveform is required to compute the modified threshold $K_{i}$, the operations needed to calculate them have very low complexity and can be easily implemented in real time, thus waveforms do not need to be stored to apply this method. There are other TOA estimation methods that generate unbiased TDOAs, as methods based on Akaike information criterion (AIC) [8,31], but these methods present higher computational complexity than the fixed threshold method (which only needs comparisons) and our TOA estimation technique, whose complexity is dominated by the calculation of the energy of the signal.

This method only works in structures where waves propagate according to (18), thus it will not be effective if hits contain wave reflections. In this case, hits must be windowed to avoid adding the energy of reflections into the energy of the hit. The proposed TOA estimation method is employed in Section 5.1 to compare the source position estimators presented in this work. The performance of these estimator using the fixed threshold method and the proposed TOA estimator are also compared.

## 5. The Optimal Source Position Estimator

In this section, we derive an expression for the optimal cost function (that is, the one that yields the minimum mean-square localization error among all possible TOA-based estimators) assuming TOAs follow a Gaussian mixture model (GMM), which is a good approximation for high sampling rate as discussed in Section 3.3. We prove that $J_{\mathrm{TOA}}$ is the optimal cost function when the noise is small and independent and identically distributed (i.i.d.) in time and across sensors. In order to determine the optimal estimator, the Fisher information matrix must be calculated and compared to the inverse of the covariance matrix of our position estimator. It is worth noting that the Fisher information matrix for passive localization problem using Gaussian TOA or TDOA measurements (that is, a Gaussian mixture with M=1 components) was calculated in [32].

We consider that TOAs follow a mixture of *M* Gaussian distributions with weights $w_{1},\cdots ,w_{M}$, covariance matrices $C_{1},\cdots ,C_{M}$ and means $v_{1},\cdots ,v_{M}$. In practice, all these parameters depend on the source position, but in order to derive the optimal estimator we assume that $C_{k}$ and $w_{k}$ are independent of the source position, while $v_{k}$ is modeled as $v_{k}=\tau \left(x\right)+b_{k}\left(x\right)+t1$, where b(x) is a TOA bias model that may depend on x, $1={\left[\begin{array}{ccc} 1 & \cdots & 1 \end{array}\right]}^{T}$, $\tau \left(x\right)={\left[\begin{array}{c} \tau_{1}\left(x\right),\cdots ,\tau_{N}\left(x\right) \end{array}\right]}^{T}$ and *t* is the instant of emission of the wave. Thus, defining the vector of measured TOAs $u={\left[\begin{array}{c} t_{1},t_{2},\cdots ,t_{N} \end{array}\right]}^{T}$, the TOA pdf is given by:

(20) $$f\left(u;x,t\right)=\sum_{k=1}^{M}\frac{w_{k}e^{-\frac{1}{2}{\left(u-v_{k}\right)}^{T}C_{k}^{-1}\left(u-v_{k}\right)}}{\sqrt{{\left(2\pi \right)}^{N}\det C_{k}}}.$$

We also assume that for each u there is only one dominant term in the sum (20), that is, there is an index m=m(u;x,t) such that for each set u of possible measured TOAs, the only term in (20) that is not approximately zero is the *m*-th component, so:

(21) $$f\left(u;x,t\right)\approx \frac{w_{m}e^{-\frac{1}{2}{\left(u-v_{m}\right)}^{T}C_{m}^{-1}\left(u-v_{m}\right)}}{\sqrt{{\left(2\pi \right)}^{N}\det C_{m}}}.$$

This is a reasonable hypothesis in the case where the sampling rate is such that there will be at least one sample below the threshold in (2) after each maximum, that is, the sampling rate should satisfy $f_{s}\gg 4f_{0}$, as in the example given in Section 3.3.

### 5.1. Optimal Source Position Estimator

Let $S_{1},S_{2},\cdots ,S_{M}$ be a partition of $R^{N}$ such that the *k*-th mixture component is different than zero for $u\in S_{k}$ and approximately zero for $u\notin S_{k}$. Each Gaussian mixture component lies in a single set $S_{k}$, thus $S_{k}$ can be viewed as the "support" of the *k*-th Gaussian component. The proposed optimal estimator defined below consists on estimating *m* such that $u\in S_{m}$ and estimating the source position with the conditional maximum likelihood estimator given that $u\in S_{m}$ (we show below how to estimate *m* even without knowing x and *t*).

The conditional probability distribution of u given that $u\in S_{k}$ is $f\left(u;x,t|u\in S_{k}\right)=\frac{1}{\sqrt{{\left(2\pi \right)}^{N}\det C_{k}}}e^{-\frac{1}{2}{\left(u-v_{k}\right)}^{T}C_{k}^{-1}\left(u-v_{k}\right)}$, thus the conditional maximum likelihood estimator given that $u\in S_{k}$ is obtained by maximizing $\log\left(f(u;x,t|u\in S_{k})\right)$, or equivalently minimizing $J_{k}\left(x,t\right)=\frac{1}{2}{\left(u-v_{k}\right)}^{T}C_{k}^{-1}\left(u-v_{k}\right)$. $J_{k}\left(x,t\right)$ can be rewritten in terms of only two variables by setting $\frac{\partial }{\partial t}J_{k}\left(x,t\right)=0$ and isolating *t* in terms of x, yielding the equivalent cost function:

(22) $$\begin{array}{cc} J_{k}\left(x\right) & =\frac{1}{2}{\left(u-v_{k}\right)}^{T}C_{k}^{-1}\left(u-v_{k}\right),\\ v_{k} & =\tau \left(x\right)+b_{k}\left(x\right)+t\left(x\right)1,\\ t(x) & =\frac{1}{N}1^{T}u-\frac{1}{N}1^{T}\left(b_{k}\left(x\right)+\tau \left(x\right)\right). \end{array}$$

Assume we could choose $k=m^{*}\overset{\mathrm{def}}{=}m\left(x^{*},t^{*}\right)$, that is, the index of the mixture component that most contributes to $f(u;x^{*},t^{*})$, where $x^{*}$, $t^{*}$ are the actual source position and instant of emission. Hence, recalling that u is the vector of measured TOAs,

(23) $$\begin{array}{cc} J_{\mathrm{opt}}\left(x\right) & =J_{m^{*}}\left(x\right),\\ m^{*} & =\arg\max_{k}\frac{w_{k}e^{-\frac{1}{2}{\left(u-v_{k}^{*}\right)}^{T}C_{k}^{-1}\left(u-v_{k}^{*}\right)}}{\sqrt{{\left(2\pi \right)}^{N}\det C_{k}}}, \end{array}$$

where $v_{k}^{*}$ is $v_{k}$ computed at the actual source position $x^{*}$, i.e., $v_{k}^{*}=E\left\{u|u\in S_{k}\right\}=\tau \left(x^{*}\right)+b_{k}+t^{*}1$. Even though $\left(x^{*},t^{*}\right)$ are unknown variables, in Section 5.3 we show how to obtain $v_{k}^{*}$ from the TOA pmf, which is estimated using the noisy signals acquired by sensors. $m^{*}$ can be correctly chosen even if an imprecise value for $v_{k}^{*}$ is used because $m^{*}$ is chosen by picking the maximum of a finite set, thus a perturbation on these values only affects $m^{*}$ if it is large enough to change the index of the maximum value.

We claim that the cost function $J_{\mathrm{opt}}\left(x\right)$ yields the optimal estimator when minimized if the noise level is not very high and the Hessian of $J_{\mathrm{opt}}\left(x\right)$ is approximately constant around the actual source position, in which case $J_{\mathrm{opt}}\left(x\right)$ can be approximated by its second-order Taylor polynomial around $x^{*}$. In order to prove this, we calculate the Fisher information matrix in Appendix A and the covariance matrix of our estimator in Appendix B. The covariance matrix (A11) coincides with the inverse of the submatrix associated with the spatial components of the Fisher information matrix (A2), which proves that the proposed estimator is indeed optimal.

It must be highlighted that we made the following hypotheses to compute the covariance matrix:

1. The noise variance is not high, so that the Taylor approximation (A3) works. This is also necessary to guarantee that the estimator is approximately unbiased.
2. The parameters of the TOA pdf $w_{k},b_{k}$ and $C_{k}$ are known.

If these conditions do not hold, (23) may not be optimal. However, we show in Section 5.3 how to obtain an approximation to the optimal estimator when the assumptions are not true, and in Section 6 we show that the resulting estimator usually outperforms other estimators.

### 5.2. The Maximum Likelihood Estimator

The Maximum likelihood estimator (MLE) is obtained by finding the values of x that maximize (20). Given the definition of m=m(x,t), this can be obtained by solving:

(24) $$\hat{x},\hat{t},m=\arg\min_{x,t,k}\frac{1}{2}{\left(u-v_{k}\right)}^{T}C_{k}^{-1}\left(u-v_{k}\right)+\kappa_{k},$$

where $\kappa_{k}=-\log\left(\frac{w_{k}}{\sqrt{{\left(2\pi \right)}^{N}\det C_{k}}}\right)$. Since $\kappa_{k}$ does not depend on x, the MLE coincides with the arguments that minimize $J_{m}\left(x\right)$ for $m=\arg\min_{k}\min_{x}J_{k}\left(x\right)+\kappa_{k}$.

Therefore, the optimal estimator may coincide with the MLE if they share the same value for *m*, but they are different estimators in general. However, their estimates always coincide if the noise is small enough so that the number of components in the Gaussian mixture is M=1, as in this case both estimators always pick m=1. Note that if M=1 and TOAs are unbiased and i.i.d, both the MLE and the optimal estimator coincide with $J_{\mathrm{TOA}}$.

### 5.3. The Gaussian Mixture TOA Estimator

In order to show that (23) is optimal, we assumed that the parameters of the Gaussian mixture are known and that *m* is such that $u\in S_{m}$, but these parameters must be estimated in practical problems. In this section, we describe a procedure to choose *m* and to estimate the mixture parameters using the noisy signals received by the sensors, leading to a suboptimal estimator we call the *Gaussian mixture TOA estimator* (GMTOA). To simplify the explanation of our method, we assume that the signals are corrupted by white noise and that the noise at different sensors is independent.

We estimate the TOA pmf for each sensor using (17), but instead of the noiseless waveform we compute $q_{n}$ using the noisy signal, that is,

(25) $$p_{i}\left[n\right]=\left(1-F_{W}\left({\hat{q}}_{n}\right)\right)\prod_{k=0}^{n-1}F_{W}\left({\hat{q}}_{k}\right),\;{\hat{q}}_{n}=K-\left|r_{i}\left[n\right]\right|.$$

Note that evaluating this pmf does not depend on previous knowledge of the source position, which we use to extract the GMM parameters. Thus, we convert the pmf $p_{i}\left[n\right]$ to a pdf $p_{i}\left(t\right)=\frac{1}{T}p_{i}\left[\mathrm{round}\left(t/T\right)\right]$ and then we fit $p_{i}\left(t\right)$ into a Gaussian mixture model, as done in Section 3.3. Then, the means $\mu_{i,1},\cdots ,\mu_{i,M_{i}}$, variances $\sigma_{i,1}^{2},\cdots ,\sigma_{i,M_{i}}^{2}$ and weights $w_{i,1},\cdots ,w_{i,M_{i}}$ are extracted for each mixture component k=1,2,⋯,M and sensor i=1,2,⋯,N. Note that, since we are assuming the noise at different sensors to be independent, the TOAs in different sensors will be independent and the full mixture model will be the product of the distributions for each sensor, and have in total $M=M_{1}M_{2}\cdots M_{N}$ components.

Now, it is necessary to choose the index $m_{i}$ in the range $1\leq m_{i}\leq M_{i}$. Since the TOAs are independent, it is possible to choose the active index $m_{i}$ for each sensor independently. Index $m_{i}$ is chosen by finding the Gaussian component that most contributes to the fitted pdf:

(26) $$m_{i}=\arg\max_{k}\frac{w_{i,k}}{\sqrt{2\pi \sigma_{i,k}^{2}}}e^{-\frac{{\left(u_{i}-\mu_{i,k}\right)}^{2}}{2\sigma_{i,k}^{2}}}.$$

Finally, the TOA bias for the *i*-th sensor $b_{i}$ must be extracted from the mean of the chosen GMM component $\mu_{m_{i}}$. Ideally, $\mu_{m_{i}}$ and $b_{m_{i}}$ would be related by $\mu_{m_{i}}=\tau_{i}\left(x^{*}\right)+t^{*}+b_{m_{i}}$, but $\tau_{i}\left(x^{*}\right)$ and $t^{*}$ are not known. As such, $b_{m_{i}}$ is estimated by approximating $\tau_{i}\left(x^{*}\right)+t^{*}$ as the first instant the estimated probability distribution $p_{i}\left[n\right]$ crosses a very small fixed threshold $K_{p}$, that is, the instant where it assumes a non-negligible value (in our simulations, we use $K_{p}=10^{-3}$). Hence, $b_{m_{i}}$ is estimated as:

(27) $$b_{m_{i}}=\mu_{m_{i}}-\bar{n}T,\;\bar{n}=\min_{n}\left\{n:\;p_{i}\left[n\right]\geq K_{\mathrm{pdf}}\right\}.$$

Therefore, the source position estimated by our proposed estimator is:

(28) $$\hat{x}=\arg\min_{x}{\left(u-v\right)}^{T}C^{-1}\left(u-v\right),$$

where v=τ(x)+b+t(x)1, $C=\mathrm{diag}(\sigma_{m_{1}}^{2},\sigma_{m_{2}}^{2},\cdots ,\sigma_{m_{N}}^{2})$, $t\left(x\right)=\frac{1}{N}1^{T}u-\frac{1}{N}1^{T}\left(b+\tau \right)$ and $b={\left[\begin{array}{ccc} b_{m_{1}} & \cdots & b_{m_{N}} \end{array}\right]}^{T}$. The proposed algorithm is summarized in Algorithm 1.

Note that our method requires the signals $r_{i}\left[n\right]$ sampled by each sensor to localize the source, thus it does not use only TOAs as the cost functions $J_{\mathrm{TOA}}$, $J_{\mathrm{TDOA}}$ and $J_{CLS}$. Storing waveforms may be an issue for long acoustic emission tests that last weeks or months, as the waveforms may require an enormous storage capacity. However, it is possible to use our method without the need to store the whole waveforms because it uses $r_{i}\left[n\right]$ only to calculate $p_{i}\left[n\right]=\left(1-F_{W}\left({\hat{q}}_{n}\right)\right)\prod_{k=0}^{n-1}F_{W}\left({\hat{q}}_{k}\right)$, which can be computed recursively: Defining $Q_{i}\left[n\right]=\prod_{k=0}^{n-1}F_{W}\left({\hat{q}}_{k}\right)$ and recalling that ${\hat{q}}_{n}=K-\left|r_{i}\left[n\right]\right|$, we have:

(29) $$\begin{array}{cc} p_{i}\left[n\right] & =\left(1-F_{W}\left({\hat{q}}_{n}\right)\right)Q_{i}\left[n\right], \end{array}$$

(30) $$\begin{array}{cc} Q_{i}\left[n\right] & =Q_{i}\left[n-1\right]F_{W}\left({\hat{q}}_{n-1}\right). \end{array}$$

Since the computational cost of computing $F_{W}\left({\hat{q}}_{n}\right)$ is small, it is possible to compute $p_{i}\left[n\right]$ in real time and discard the signals $r_{1}\left[n\right],\cdots ,r_{N}\left[n\right]$ instead of storing them. Storing $p_{i}\left[n\right]$ instead of the signals is much more efficient because $p_{i}\left[n\right]$ assumes a non-negligible value for only a small number of samples, thus it is possible to store only these non-negligible samples. Therefore, our method does not present the problem of storing a huge number of waveforms in long acoustic emission tests as methods that explicitly require the sampled signals as [33,34,35].
**Algorithm 1:** GMTOA source position estimator
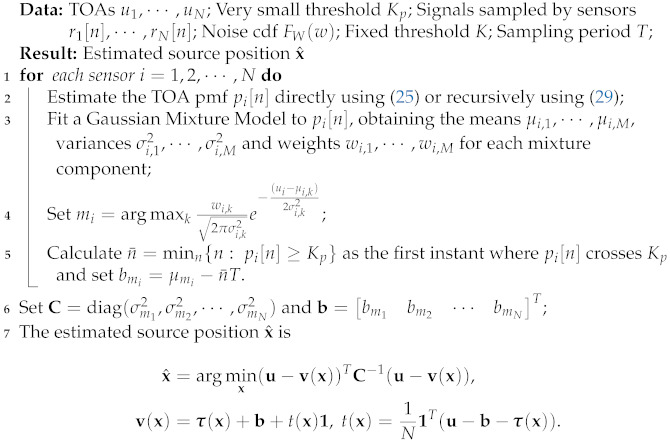


## 6. Simulations

In this section, we assess the performance of our proposed source position estimator through simulations. Four sensors were positioned at the coordinates (0,0),(0,1),(1,0) and (1,1) in order to estimate the position of sources located inside the convex polygon whose vertices are the positions of the sensors (in this case, this polygon is a square) in several scenarios. In all cases, the cost functions were minimized using the mean position of the sensors (0.5,0.5) as initial condition, except for the MLE which was implemented by finding the global maximum of the exact Gaussian mixture pdf (20) using *M* different initial conditions, each one obtained by minimizing $J_{k}\left(x\right)$ for k=1,2,⋯,M.

### 6.1. Unbiased i.i.d. Gaussian TOAs, Different Source Positions

In order to test the optimality of our algorithm in ideal conditions, we generated TOAs as Gaussian random variables with no bias and standard deviation of 1μs and 10μs. Denoting the source position as $x^{*}={\left[\begin{array}{cc} x & y \end{array}\right]}^{T}$, *y* was fixed at y=0.4m and *x* was swept from 0 to 1. For each position, the Mean Square Error (MSE) of the estimators was calculated using $10^{5}$ samples and used to compute the efficiency of the estimator, defined as $\eta =\frac{\mathrm{tr}\{I{\left(x^{*}\right)}^{-1}\}}{\mathrm{tr}\{E\left\{\left(\hat{x}-x^{*}\right){\left(\hat{x}-x^{*}\right)}^{T}\right\}\}}$. Note that η=1 if the estimator is optimal.

**Figure 3:**
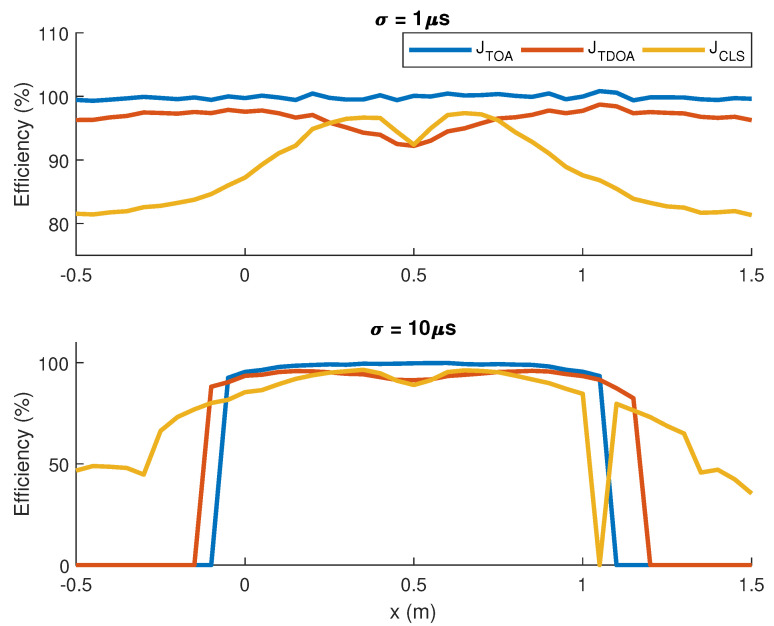
Efficiency of the source position estimators in terms of the *x* coordinate of the actual source position (x,0.4) for unbiased Gaussian TOAs with standard deviation σ=1μs (**top**) and σ=10μs (**bottom**).

The efficiency in terms of the source position is shown in Figure 3. For σ=1μs, the efficiency of the cost function $J_{\mathrm{TOA}}$ is very close to 100%, while $J_{\mathrm{TDOA}}$ and $J_{CLS}$ show worse performance for all source positions, indicating that $J_{\mathrm{TOA}}$ is indeed the optimal estimator for unbiased i.i.d. Gaussian TOAs.

On the other hand, for σ=10μs, the estimates obtained through $J_{\mathrm{TOA}}$ are very close to optimal when the source lies inside the region of interest (the convex polygon whose vertexes are the positions of the sensors). However, outside this polygon, $J_{\mathrm{TOA}}$ is no longer the optimal cost function because the approximations used to prove the estimator is optimal are not precise in this region. This happens because the Taylor approximation (A3) does not hold when $x^{*}$ is in a region where the Hessian of $J_{\mathrm{TOA}}\left(x\right)$ is not approximately constant, which may happen if the source is close to a sensor.

Even though $J_{\mathrm{CLS}}$ has worse accuracy than $J_{\mathrm{TOA}}$ in most cases, its efficiency does not fall down abruptly for *x* outside the region of interest, especially in the case of high noise level, in which the performance of the other methods is close to zero outside the region between sensors.

It is worth noting that in practice the sensors are scattered around the monitored region, thus the precision of localization algorithms is more important for sources lying inside the region of interest, in which case $J_{\mathrm{TOA}}$ performs better than the other methods for both noise variances.

### 6.2. Fixed Source Position and Different SNRs

Next, we performed simulations estimating the TOAs from noisy signals, as happens in practice. Note that:

- For low SNR, the Taylor approximation (A3) (Appendix B) does not hold and the estimator may be biased.
- The MSE of GMTOA depends on the quality of the estimated GMM parameters, being optimal only if these estimates match the actual ones.
- The GMM assumption for TOAs is an approximation, so an estimator may show lower MSE than the derived CRLB when empirical TOAs are used.

We have done four simulations in similar conditions as before, except that the source position is fixed at $x^{*}={\left[\begin{array}{cc} 0.3 & 0.4 \end{array}\right]}^{T}$. In all simulations, hits were generated according to (18) using $a_{i}=\frac{e^{-\alpha d_{i,s}}}{\sqrt{d_{i,s}}}$ and $\psi \left(nT\right)=\sin^{2}\left(\frac{\pi n}{1000}\right)\sin\left(\frac{2\pi n}{67}\right)$ (a modulated von Hann window), with white Gaussian noise of different variances. The sampling rate is 10Msps, hence the frequency of the carrier is $\frac{10^{7}}{67}\approx 150\;\mathrm{kHz}$, the resonance frequency for some acoustic emission sensors.

In these simulations, TOAs were calculated using the TOA estimation technique proposed in Section 4 with a fixed threshold K=0.3. The GMTOA estimator was implemented using Algorithm 1, and the parameters obtained by this algorithm were used in the MLE.

#### 6.2.1. TOAs Following a GMM and Known Parameters

In this simulation, the noisy hits were used to obtain auxiliary TOAs through the fixed threshold method. Then, the mean and variance of these auxiliary TOAs were computed and used to generate TOAs following a Gaussian mixture distribution with known parameters. Note that $v_{k}$ is not known because it depends on the tentative source position.

**Figure 4:**
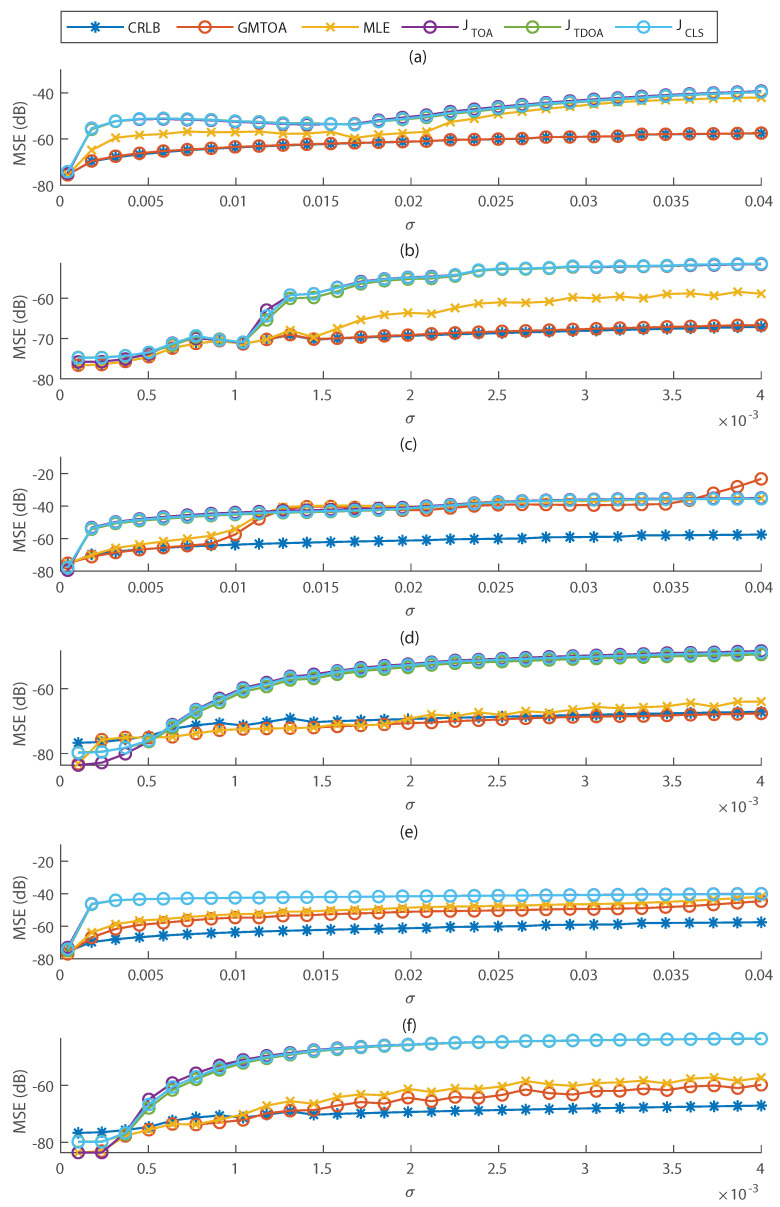
MSE of TOA–based localization methods in terms of noise standard deviation in several scenarios. (**a**,**b**) The parameters of Gaussian mixtures used by GMM and optimal estimator are the actual ones, and TOAs are distributed according to a GMM. (**c**,**d**) TOAs and the GMM parameters are estimated from noisy hits. (**e**,**f**) TOAs and the GMM parameters are estimated from hits filtered by a low-pass filter, in which case noise is self-correlated. (**b**,**d**,**f**) are, respectively, zoomed versions of (**a**,**c**,**e**) at smaller variances.

The MSE in terms of noise standard deviation for the presented methods is shown in Figure 4a,b. The MSE of the GMTOA estimator is very close to the CRLB for all noise variances, confirming our derivations. Moreover, the maximum likelihood estimator does not coincide with the optimal one if σ is high, but the MLE is optimal for low noise level.

#### 6.2.2. Empirical TOAs and Unknown Parameters

In this simulation, the actual TOAs obtained from the fixed threshold method are used to localize the source. Hence, TOAs are not exactly distributed according to a Gaussian mixture in this case. The Cramér–Rao lower bound was calculated based on the parameters estimated by the GMTOA Estimator from noisy hits using Algorithm 1.

Figure 4c,d shows that if the noise variance is very high, the GMTOA estimator does not perform better than the others, but it does have a significantly better performance for intermediate variances where the TOA pdfs cannot be approximated as single Gaussian distributions.

On the other hand, the proposed estimator has lower MSE than the other estimators for very low variances, but it may perform worse than them if the variance is not very low, but low enough so that the TOA pdf is nearly Gaussian. This is because the MSE of $J_{\mathrm{TOA}}$, $J_{\mathrm{TDOA}}$ and $J_{CLS}$ fall abruptly when the TOA distribution becomes approximately Gaussian (or a mixture with only one component), while the MLE and the GMTOA estimator do not because they depend on the noisy parameters of the estimated Gaussian pdf. For smaller variances, the parameters from the pdf approximate well the actual ones, thus the GMTOA estimator becomes again better than $J_{\mathrm{TOA}}$ and $J_{\mathrm{TDOA}}$.

Note that the MSEs of the estimators fall below the Cramér–Rao lower bound if σ is small. This happens because we derived the CRLB for TOAs following a known Gaussian mixture distribution, but the true distribution is discrete, not exactly a mixture of Gaussians.

#### 6.2.3. TOAs Obtained from Filtered Hits and Unknown Parameters

The main issue with the maximum likelihood and GMTOA estimators is that they depend on the noise pdf, which is not accessible in practice and must be estimated. If the noise variance is large, these parameters can be poorly estimated and cause performance loss in the localization methods, as verified in the last simulation. To test the robustness of these estimators in a scenario of non-white noise, a lowpass filter is applied to hits before calculating the threshold in this simulation. The filter used was a Butterworth filter of order 15 and digital cutoff frequency $\omega_{c}=0.1\pi$ (or 1 MHz), but the TOA pmf was calculated assuming white Gaussian noise.

The results are shown in Figure 4e,f. With the filter, the parameters of the GMM are better estimated, and the GMTOA estimator has better performance than all other methods even for high variance. The MLE also presents much lower MSE than $J_{\mathrm{TOA}}$ and $J_{\mathrm{TDOA}}$ for high noise level.

Since in this simulation GMTOA assumes white noise to calculate the TOA pmf (which is used to extract the GMM parameters), it may have worse performance using filtered hits (which are corrupted by non-white noise) instead of the original hits for some values of σ, but in most cases using filtered hits implied in lower MSE.

#### 6.2.4. Using Biased TDOAs

**Figure 5:**
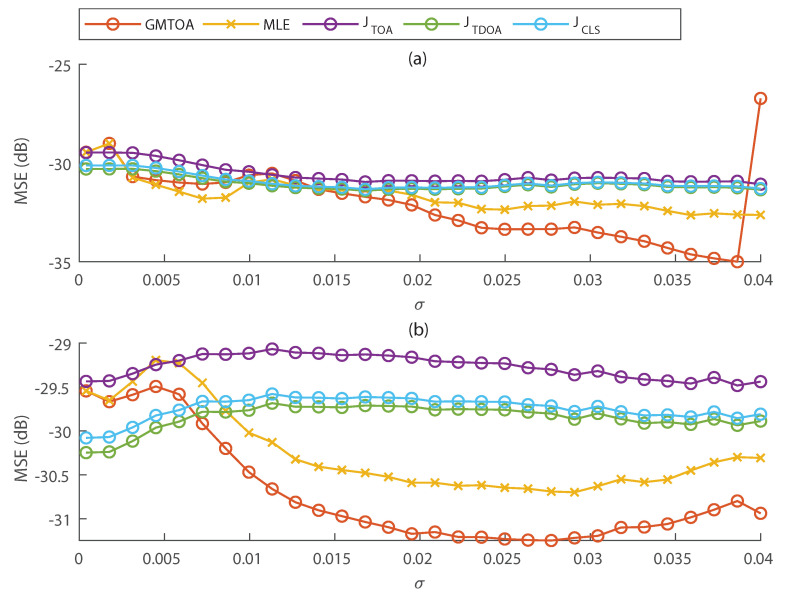
Comparison of localization methods using TOAs extracted from the original noisy hits (**a**) or hits filtered by a low–pass filter (**b**). TOAs were obtained with the fixed threshold method, yielding biased TDOAs due to attenuation.

In all previous simulations, TOAs were obtained using a proposed modification of the fixed threshold method described in Section 4, which generates unbiased TDOAs. Figure 5 shows a simulation with the same setup as in Section 6.2.2 and Section 6.2.3, but the fixed threshold method was used to obtain TOAs instead of the proposed modification. As such, TDOAs are biased in this simulation.

Comparing Figure 4c,e with Figure 5, the proposed TOA estimation method significantly decreases the MSE of TOA-based localization methods. These figures also show that the GMTOA estimator achieves better performance than other methods in most cases for TOAs obtained from original hits and in all cases if hits are filtered before TOA extraction.

It is worth noting that if biased TOAs are used directly for estimation, the MSE may decrease for higher noise variance because bias may be reduced.

## 7. Conclusions

In this work, we derived the expression for the TOA pmf obtained through the threshold method, and showed that it is not Gaussian-distributed as usually assumed. We show that the TOA pmf can only be approximated as a Gaussian distribution if the sampling rate is high and the noise level is small. The distribution can be modeled by a mixture of Gaussian distributions for higher noise level. We also presented a TOA estimation method based on the fixed threshold algorithm that generates unbiased TDOAs in structures where hits are approximately delayed and attenuated versions of each other.

The optimal source position estimator was derived assuming TOAs follow a mixture of Gaussian distributions of known parameters. This estimator coincides with the maximum likelihood estimator for small noise level, in which case TOAs are Gaussian. Since the mixture parameters are not known in practical applications, we proposed a method to estimate them from noisy waveforms, resulting in the GMTOA estimator, a suboptimal method to localize the source. We verified in simulation that GMTOA yields better results than other methods for both biased and unbiased TDOAs in most cases, even if the TOA pmf is not exactly a mixture of Gaussian distributions, and its performance is enhanced if a low-pass filter is applied to hits before extracting TOAs.

Even though the proposed estimator performed better than other methods in simulation, it is necessary to further validate it with data collected from an acoustic emission test. Further work also includes extending our TOA estimation method to generate unbiased TDOAs for more complex propagation models, in particular for structures where hits contain many reflections of the wave emitted by the source.

## Abbreviations

The following abbreviations are used in this manuscript:

TOA	Time of arrival
TDOA	Time difference of arrival
pdf	Probability density function
cdf	Cumulative density function
pmf	Probability mass function
GMM	Gaussian mixture model
GMTOA	Our proposed time of arrival estimator based on Gaussian mixtures
CLS	Constrained least squares
MLE	Maximum likelihood estimator
CRLB	Cramér–Rao lower bound
MSE	Mean square error
SNR	Signal-to-noise ratio

## Appendix A. Derivation of the Fisher Information Matrix

The log-likelihood function is L(u;x,t)=log(f(u;x,t)), where f(u;x,t) is given by (21). In order to shorten the next equations, let us define the auxiliary variable ${\bar{\tau }}_{m}=\tau \left(x^{*}\right)+b_{m}\left(x^{*}\right)$. Thus, the first and second derivatives of L(x,t) calculated at the actual source position $x^{*}$ and instant of emission $t^{*}$ are: $L_{x}={\bar{\tau }}_{m,x}^{T}C_{m}^{-1}\left(u-{\bar{\tau }}_{m}-t^{*}1\right)$, $L_{y}={\bar{\tau }}_{m,y}^{T}C_{m}^{-1}\left(u-{\bar{\tau }}_{m}-t^{*}1\right)$, $L_{t}=1^{T}C_{m}^{-1}\left(u-{\bar{\tau }}_{m}-t^{*}1\right)$, $L_{xx}={\bar{\tau }}_{m,xx}^{T}C_{m}^{-1}\left(u-{\bar{\tau }}_{m}-t^{*}1\right)-{\bar{\tau }}_{m,x}^{T}C^{-1}{\bar{\tau }}_{m,x}$, $L_{yy}={\bar{\tau }}_{m,yy}^{T}C_{m}^{-1}\left(u-{\bar{\tau }}_{m}-t^{*}1\right)-{\bar{\tau }}_{m,y}^{T}C^{-1}{\bar{\tau }}_{m,y}$, $L_{tt}=-1^{T}C_{m}^{-1}1$, $L_{xy}={\bar{\tau }}_{m,xy}^{T}C_{m}^{-1}\left(u-{\bar{\tau }}_{m}-t^{*}1\right)-{\bar{\tau }}_{m,x}^{T}C^{-1}{\bar{\tau }}_{m,y}$, $L_{xt}=-1^{T}C_{m}^{-1}{\bar{\tau }}_{m,x}$, $L_{yt}=-1^{T}C_{m}^{-1}{\bar{\tau }}_{m,y}$.

The Fisher information matrix $I\left(x^{*},t^{*}\right)=-E\left\{\left[\begin{array}{ccc} L_{xx} & L_{xy} & L_{xt}\\ L_{xy} & L_{yy} & L_{yt}\\ L_{xt} & L_{yt} & L_{tt} \end{array}\right]\right\}$ can be decomposed into:

(A1) $$I\left(x^{*},t^{*}\right)=\sum_{k=1}^{M}w_{k}E\left\{\left[\begin{array}{ccc} L_{xx} & L_{xy} & L_{xt}\\ L_{xy} & L_{yy} & L_{yt}\\ L_{xt} & L_{yt} & L_{tt} \end{array}\right]\;|\;u\in S_{k}\right\}.$$

Each conditional expectation being summed in (A1) can be computed by assuming u follows a Gaussian distribution with mean $v_{k}^{*}=\tau_{k}\left(x^{*}\right)+b_{k}\left(x^{*}\right)-t^{*}1$ and covariance matrix $C_{k}$. Applying each conditional expectation to $L_{xx}$, $L_{xy}$ and $L_{yy}$, and substituting the results in (A1), we conclude that the Fisher information matrix $I(x^{*},t^{*})$ is given by:

(A2) $$\sum_{k=1}^{M}w_{k}\left[\begin{array}{ccc} {\bar{\tau }}_{k,x}^{T}C_{k}^{-1}{\bar{\tau }}_{k,x} & {\bar{\tau }}_{k,x}^{T}C_{k}^{-1}\tau_{k,y} & 1^{T}C_{k}^{-1}{\bar{\tau }}_{k,x}\\ {\bar{\tau }}_{k,y}^{T}C_{k}^{-1}{\bar{\tau }}_{k,y} & {\bar{\tau }}_{k,x}^{T}C_{k}^{-1}{\bar{\tau }}_{k,y} & 1^{T}C_{k}^{-1}{\bar{\tau }}_{k,y}\\ 1^{T}C_{k}^{-1}{\bar{\tau }}_{k,x} & 1^{T}C_{k}^{-1}{\bar{\tau }}_{k,y} & 1^{T}C_{k}^{-1}1. \end{array}\right]$$

## Appendix B. Derivation of the Covariance Matrix for the Optimal Estimator

In order to calculate the covariance matrix of the estimator $\hat{x}=\arg\min_{x}J\left(x\right)$, where J(x) is given by (23), we approximate J(x) by its second-order Taylor polynomial around the source position $x^{*}$:

(A3) $$J\left(x\right)\approx J\left(x^{*}\right)+\nabla J\left(x^{*}\right)\left(x-x^{*}\right)+\frac{1}{2}{\left(x-x^{*}\right)}^{T}H\left(x^{*}\right)\left(x-x^{*}\right).$$

Setting ∇J(x)=0 yields $x-x^{*}\approx -H^{-1}\left(x^{*}\right)\nabla J\left(x^{*}\right)$, thus:

(A4) $$\left(x-x^{*}\right){\left(x-x^{*}\right)}^{T}\approx H^{-1}\left(x^{*}\right)\nabla J\left(x^{*}\right)\nabla J{\left(x^{*}\right)}^{T}H^{-1}\left(x^{*}\right).$$

To calculate the bias and the covariance matrix of the estimated source position, we use the following approximations:

(A5) $$E\left\{x-x^{*}\right\}\approx -E{\left\{H\left(x^{*}\right)\right\}}^{-1}E\left\{\nabla J\left(x^{*}\right)\right\}$$

and

(A6) $$E\left\{\left(x-x^{*}\right){\left(x-x^{*}\right)}^{T}\right\}\approx E{\left\{H\left(x^{*}\right)\right\}}^{-1}E\left\{\nabla J\left(x^{*}\right)\nabla J{\left(x^{*}\right)}^{T}\right\}E{\left\{H\left(x^{*}\right)\right\}}^{-1}.$$

These approximations are also used in [28,36] to derive the optimal estimator for the source localization problem in the scenario where the instant the source emits a signal is known, as in radar applications.

The first and second partial derivatives of J(x) with respect to *x* and *y* are: $J_{x}=2v_{m,x}^{*T}C_{m}^{-1}\left(v_{m}^{*}-u\right)$, $J_{y}=2v_{m,y}^{*T}C_{m}^{-1}\left(v_{m}^{*}-u\right)$, $J_{xx}=2v_{m,xx}^{*T}C_{m}^{-1}\left(v_{m}^{*}-u\right)+2v_{m,x}^{*T}C_{m}^{-1}v_{m,x}^{*}$, $J_{yy}=2v_{m,yy}^{*T}C_{m}^{-1}\left(v_{m}^{*}-u\right)+2v_{m,y}^{*T}C_{m}^{-1}v_{m,y}^{*}$ and $J_{xy}=2v_{m,xy}^{*T}C_{m}^{-1}\left(v_{m}^{*}-u\right)+2v_{m,x}^{*T}C_{m}^{-1}v_{m,y}^{*}.$

Using the derivatives calculated above, we obtain:

(A7) $$\nabla J\left(x^{*}\right)=2\left[\begin{array}{c} v_{m,x}^{*T}C_{m}^{-1}\left(v_{m}^{*}-u\right)\\ v_{m,y}^{*T}C_{m}^{-1}\left(v_{m}^{*}-u\right) \end{array}\right],$$

(A8) $$\begin{array}{cc} E\{\nabla J\left(x^{*}\right)\nabla J{\left(x^{*}\right)}^{T}\} & =\sum_{k=1}^{M}w_{k}E\left\{\nabla J\left(x^{*}\right)\nabla J{\left(x^{*}\right)}^{T}\;|\;u\in S_{k}\right\}\\ & =4\sum_{k=1}^{M}w_{k}\left[\begin{array}{cc} v_{k,x}^{T}C_{k}^{-1}v_{k,x} & v_{k,x}^{T}C_{k}^{-1}v_{k,y}\\ v_{k,x}^{T}C_{k}^{-1}v_{k,y} & v_{k,y}^{T}C_{k}^{-1}v_{k,y} \end{array}\right] \end{array}$$

and

(A9) $$E\left\{H\left(x^{*}\right)\right\}=2\sum_{k=1}^{M}w_{k}\left[\begin{array}{cc} v_{k,x}^{T}C_{k}^{-1}v_{k,x} & v_{k,x}^{T}C_{k}^{-1}v_{k,y}\\ v_{k,x}^{T}C_{k}^{-1}v_{k,y} & v_{k,y}^{T}C_{k}^{-1}v_{k,y} \end{array}\right].$$

As $E\left\{u|u\in S_{k}\right\}=v_{k}$, we have from (A7) that:

(A10) $$E\left\{\nabla J\left(x^{*}\right)\right\}=2\sum_{k=1}^{M}w_{k}E\left\{\left[\begin{array}{c} v_{k,x}^{T}C_{k}^{-1}\left(v_{k}-u\right)\\ v_{k,y}^{T}C_{k}^{-1}\left(v_{k}-u\right) \end{array}\right]|\;u\in S_{k}\right\}=0,$$

thus we conclude from (A5) that the position estimator is approximately unbiased: $E\left\{x-x^{*}\right\}\approx -E{\left\{H\left(x^{*}\right)\right\}}^{-1}E\left\{\nabla J\left(x^{*}\right)\right\}=0.$ In order to shorten the next expressions, let us define $A_{k}=\left[\begin{array}{cc} v_{k,x}^{T}C_{k}^{-1}v_{k,x} & v_{k,x}^{T}C_{k}^{-1}v_{k,y}\\ v_{k,x}^{T}C_{k}^{-1}v_{k,y} & v_{k,y}^{T}C_{k}^{-1}v_{k,y} \end{array}\right]$ such that $E\left\{H\left(x^{*}\right)\right\}=2\sum_{k=1}^{M}w_{k}A_{k}$ and $E\{\nabla J\left(x^{*}\right)\nabla J{\left(x^{*}\right)}^{T}\}$$=4\sum_{k=1}^{M}w_{k}A_{k}$.

From (A6), (A8) and (A9), the covariance matrix of the estimated position is $C_{x}=E\left\{\left(x-x^{*}\right){\left(x-x^{*}\right)}^{T}\right\}\approx {\left(\sum_{k=1}^{M}w_{k}A_{k}\right)}^{-1}$. Since $v_{k}={\bar{\tau }}_{k}+t1$, we have $v_{k,x}={\bar{\tau }}_{k,x}$ and $v_{k,y}={\bar{\tau }}_{k,y}$, thus:

(A11) $$C_{x}\approx {\left(\sum_{k=1}^{M}w_{k}\left[\begin{array}{cc} {\bar{\tau }}_{k,x}^{T}C_{k}^{-1}{\bar{\tau }}_{k,x} & {\bar{\tau }}_{k,x}^{T}C_{k}^{-1}{\bar{\tau }}_{k,y}\\ {\bar{\tau }}_{k,y}^{T}C_{k}^{-1}{\bar{\tau }}_{k,y} & {\bar{\tau }}_{k,x}^{T}C_{k}^{-1}{\bar{\tau }}_{k,y} \end{array}\right]\right)}^{-1}.$$

This is the inverse of the submatrix obtained by selecting only the spatial components of the Fisher Information Matrix (A2). Hence, (23) is the optimal TOA-based source position estimator.

## Appendix C. Fitting p[n] into a Mixture of Gaussians

In order to implement Algorithm 1, we have to obtain the parameters of the Gaussian mixture by fitting $p_{i}\left[n\right]$ into a mixture of Gaussian distributions. Any fitting technique can be employed, but we use a simple approach that requires low computational complexity.

First, we interpolate $p_{i}\left[n\right]$ into a continuous pdf $p_{i}\left(t\right)$, which is then fitted into a Gaussian mixture model. Then, the number *M* of Gaussian components in the mixture is calculated as well as the supports $S_{i,1},\cdots ,S_{i,M_{i}}$ for each Gaussian, and finally we use $p_{i}\left(t\right)$ and the intervals $S_{i,1},\cdots ,S_{i,M_{i}}$ to compute the parameters of each mixture component, as we detail now.

Since all measured TOAs are multiple of the sampling instant, the difference between the measured TOAs and the TOAs that would be measured in continuous-time (that is, with infinite sampling rate) is modeled as a uniform distribution in the interval [−T,+T]. Hence, the pmf $p_{i}\left[n\right]$ can be interpolated into a pdf $p_{i}\left(t\right)$ given by:

(A12) $$p_{i}\left(t\right)=\sum_{n}p_{i}\left[n\right]1_{\left[nT-T/2,nT+T/2\right]}\left(t\right).$$

Next, the number $M_{i}$ of Gaussian components in the mixture and the supports $S_{i,1},S_{i,1},\cdots ,S_{i,M_{i}}$ of each mixture component are estimated, and then the parameters are extracted from each mixture component. To obtain the supports, we extract a binary signal $d_{i}\left[n\right]$ from $p_{i}\left[n\right]$ such that for each *n*, $d_{i}\left[n\right]=1$ if *n* belongs to the support of a Gaussian component. More specifically, $d_{i}\left[n\right]$ is defined as 1 if $p\left[\ell \right]\geq K_{p}\;\forall \ell \in \left[n-N_{\mathrm{gap}},n+N_{\mathrm{gap}}\right]$, and 0 otherwise, where $K_{p}$ is the small threshold used in Algorithm 1, and $N_{\mathrm{gap}}$ is a predefined integer that controls the minimum gap between two mixture components. Setting $N_{\mathrm{gap}}>0$ avoids overestimating the number of mixture components. In our simulations, we used $N_{\mathrm{gap}}=2$ samples. The supports $S_{i,1},\cdots ,S_{i,M_{i}}$ are the $M_{i}$ intervals where $d_{i}\left[n\right]=1$.

The parameters $w=(w_{i,1},w_{i,2},\cdots ,w_{i,M})$, $\mu =(\mu_{i,1},\mu_{i,2},\cdots ,\mu_{i,M})$ and $\sigma =(\sigma_{i,1}^{2},\sigma_{i,2}^{2},\cdots ,\sigma_{i,M}^{2})$ of fitted pdf ${\hat{p}}_{i}\left(t;w,\mu ,\sigma \right)$ are calculated by minimizing the Kullback–Leibler divergence [37] (which is a measure of distance between two probability distributions) between $p_{i}\left(t\right)$ and ${\hat{p}}_{i}\left(t;w,\mu ,\sigma \right)$. Thus, (w,μ,σ) is given by:

(A13) $$\arg\min_{\left(w,\mu ,\sigma \right)}\int_{-\infty }^{+\infty }p_{i}\left(t\right)\log\left(\frac{p(t)}{{\hat{p}}_{i}\left(t;w,\mu ,\sigma \right)}\right)dt=\arg\max_{\left(w,\mu ,\sigma \right)}\int_{-\infty }^{+\infty }p_{i}\left(t\right)\log\left({\hat{p}}_{i}\left(t;w,\mu ,\sigma \right)\right)dt.$$

Expanding the logarithm, disregarding the constant term and defining:

(A14) $$f_{i,m}\left(t\right)=\frac{w_{i,m}}{\sqrt{2\pi \sigma_{i,m}^{2}}}e^{-\frac{{\left(t-\mu_{i,m}\right)}^{2}}{2\sigma_{i,m}^{2}}},$$

we obtain the equivalent cost function:

(A15) $$J_{\mathrm{fit}}\left(w,\mu ,\sigma \right)=-\sum_{m=1}^{M}\int_{S_{i,m}}p_{i}\left(t\right)\log\left(f_{i,m}\left(t\right)\right)dt.$$

The parameters are estimated by equating the derivative of $J_{\mathrm{fit}}\left(w,\mu ,\sigma \right)$ with respect to each parameter to zero, substituting (A12) in $p_{i}\left(t\right)$:

(A16) $$\left[\begin{array}{c} w_{i,m}\\ \mu_{i,m}\\ \sigma_{i,m}^{2} \end{array}\right]=\sum_{nT\in S_{i,m}}p_{i}\left[n\right]\left[\begin{array}{c} 1\\ nT\\ \beta_{i,m}\left[n\right] \end{array}\right],$$

where $\beta_{i,m}\left[n\right]={\left(nT-\mu_{i,m}+\frac{T}{2}\right)}^{3}-{\left(nT-\mu_{i,m}-\frac{T}{2}\right)}^{3}$.
